# Structure of nanocrystalline calcium silicate hydrates: insights from X-ray diffraction, synchrotron X-ray absorption and nuclear magnetic resonance

**DOI:** 10.1107/S1600576716003885

**Published:** 2016-04-12

**Authors:** Sylvain Grangeon, Francis Claret, Cédric Roosz, Tsutomu Sato, Stéphane Gaboreau, Yannick Linard

**Affiliations:** a D3E/SVP, BRGM (French Geological Survey), 3 avenue Claude Guillemin, Orléans, 45060, France; b Scientific Division, Andra, 1–7 rue Jean Monnet, Parc de la Croix Blanche, Châtenay-Malabry, France; c Laboratory of Environmental Geology, Research Group of Geoenvironmental/Engineering Division of Solid Waste, Resources and Geoenvironmental/Engineering Graduate School of Engineering, Hokkaido University, Kita 13 Nishi 8, Sapporo, Japan; d Centre de Meuse/Haute Marne, Andra, Bure, 55290, France

**Keywords:** calcium silicate hydrates, C–S–H, X-ray diffraction, ^29^Si NMR, synchrotron X-ray absorption

## Abstract

The structure of nanocrystalline calcium silicate hydrates (C–S–H) having Ca/Si ratios ranging between 0.57 ± 0.05 and 1.47 ± 0.04 was studied using an electron probe micro-analyser, powder X-ray diffraction, ^29^Si magic angle spinning NMR, and Fourier-transform infrared and synchrotron X-ray absorption spectroscopies. All samples can be described as nanocrystalline and defective tobermorite. At low Ca/Si ratio, the Si chains are defect free and the Si *Q* ^3^ and *Q* ^2^ environments account, respectively, for up to 40.2 ± 1.5% and 55.6 ± 3.0% of the total Si, with part of the *Q* ^3^ Si being attributable to remnants of the synthesis reactant. As the Ca/Si ratio increases up to 0.87 ± 0.02, the Si *Q* ^3^ environment decreases down to 0 and is preferentially replaced by the *Q* ^2^ environment, which reaches 87.9 ± 2.0%. At higher ratios, *Q* ^2^ decreases down to 32.0 ± 7.6% for Ca/Si = 1.38 ± 0.03 and is replaced by the *Q* ^1^ environment, which peaks at 68.1 ± 3.8%. The combination of X-ray diffraction and NMR allowed capturing the depolymerization of Si chains as well as a two-step variation in the layer-to-layer distance. This latter first increases from ∼11.3 Å (for samples having a Ca/Si ratio <∼0.6) up to 12.25 Å at Ca/Si = 0.87 ± 0.02, probably as a result of a weaker layer-to-layer connectivity, and then decreases down to 11 Å when the Ca/Si ratio reaches 1.38 ± 0.03. The decrease in layer-to-layer distance results from the incorporation of interlayer Ca that may form a Ca(OH)_2_-like structure, nanocrystalline and intermixed with C–S–H layers, at high Ca/Si ratios.

## Abstract 2

The structure of nanocrystalline calcium silicates hydrates (C–S–H) having Ca/Si ratios ranging between 0.57 ± 0.05 and 1.47 ± 0.04 was studied. All samples are nanocrystalline and defective tobermorite. An increase of the Ca/Si ratio resulting from omission of bridging Si in the Si chains and incorporation of interlayer Ca was observed.

## 1. Introduction   

**Figure 1:**
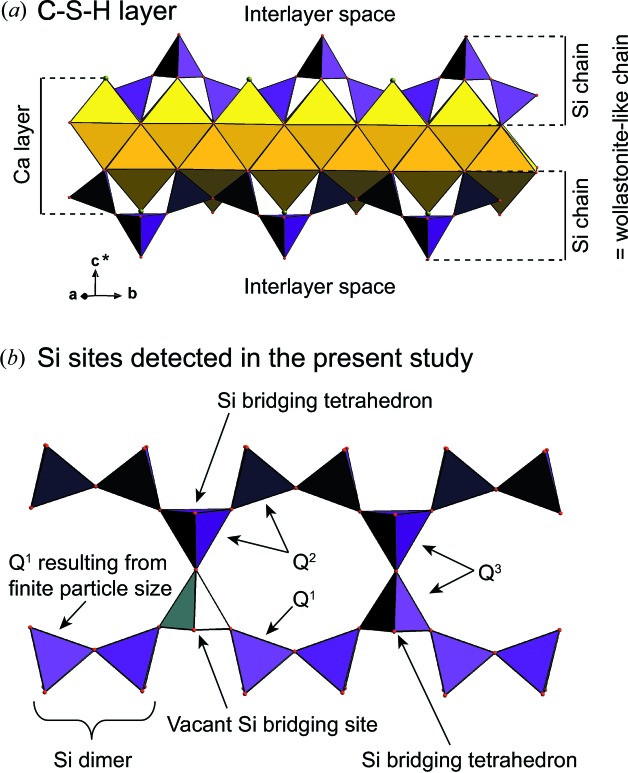
Sketch of the structure of C–S–H, in the tobermorite-like assumption. (*a*) is a view of the layer, with yellow and purple polyhedra representing, respectively, layer Ca and layer Si coordination spheres. The ribbons of Si tetrahedra, parallel to **b**, are termed the wollastonite-like chains. They are detailed in (*b*), where all Si and vacant sites identified in the present study are assigned (see text for details).

Nanocrystalline calcium silicate hydrate (C–S–H) is a synthetic phase forming the main hydration product of many types of cements (Richardson, 1999 ▸, 2008 ▸), including ordinary Portland cement. It has a complex chemistry, which manifests itself by the variability of its calcium to silicon (Ca/Si) atomic ratio, generally reported to vary between ∼0.6 and ∼2.4 (Richardson, 1999 ▸), and by its capacity to incorporate foreign elements such as aluminium or sodium (*e.g.* Bach *et al.*, 2013 ▸; Faucon *et al.*, 1998 ▸, 1999 ▸; Pardal *et al.*, 2012 ▸). C–S–H is ubiquitous in building materials, where it controls the main cement chemical (Blanc *et al.*, 2010 ▸) and mechanical (Manzano *et al.*, 2007 ▸; Pellenq *et al.*, 2009 ▸) properties. C–S–H has been the subject of many studies that aimed to build structure models which can be used to understand C–S–H mechanical properties (Abdolhosseini Qomi *et al.*, 2014 ▸) and for chemical thermodynamic modelling (Myers *et al.*, 2013 ▸, 2014 ▸; Walker *et al.*, 2007 ▸). However, the determination of the structure of C–S–H has long been hampered by the fact that it is nanocrystalline, has disordered structure (Grangeon, Claret, Linard & Chiaberge, 2013 ▸; Skinner *et al.*, 2010 ▸; Soyer-Uzun *et al.*, 2012 ▸) and is often intermixed with Ca(OH)_2_ which may be structurally bound (Chen *et al.*, 2010 ▸). These characteristics, together with the use of several different methods of analysis by different research groups, have led to the development of numerous structural models, with two of them being dominant. In the first model, the evolution of the C–S–H structure as a function of its Ca/Si ratio is described by the existence of two phases having crystal structures close either to tobermorite or to jennite (Richardson, 2008 ▸; Taylor, 1986 ▸), depending on the Ca/Si ratio. The former is assumed to be analogous to C–S–H for Ca/Si ratios lower than ∼1.3 and the latter is assumed to be analogous to C–S–H for higher Ca/Si ratios. These two minerals are layered structures built of Ca polyhedra (in sevenfold coordination in tobermorite, sixfold in jennite) with ribbons of wollastonite-like Si chains running at the surface. In both cases, the layers are separated by a hydrated interlayer space that may contain cations. In the alternative model, the whole range of Ca/Si is described using tobermorite and a varying amount of calcium hydroxide (CH), which may be structurally bound to the tobermorite layers (Richardson, 2008 ▸, 2014 ▸). C–S–H and CH form a nanocomposite (*i.e*. intimate mix of the two phases), with CH filling the micropores in the C–S–H structure, possibly through interstratification of C–S–H and CH layers (Girão *et al.*, 2010 ▸; Grangeon, Claret, Linard & Chiaberge, 2013 ▸; Richardson, 2014 ▸), when the Ca/Si ratio approaches ∼1.5. At higher Ca/Si, C–S–H and CH form a microcomposite, with CH precipitating outside C–S–H micropores (Chen *et al.*, 2010 ▸), as supported by the frequent observation of a discrete CH phase (portlandite) in X-ray diffraction patterns of C–S–H having a Ca/Si ratio higher than ∼1.5 (Garbev, Beuchle *et al.*, 2008 ▸; Renaudin *et al.*, 2009 ▸). The tobermorite-like model explains electrophoretic measurements made on C–S–H suspensions (Churakov *et al.*, 2014 ▸) and aluminium uptake by C–S–H (*e.g.* Andersen *et al.*, 2003 ▸; Myers *et al.*, 2013 ▸, 2015 ▸; Pardal *et al.*, 2012 ▸; Pegado *et al.*, 2014 ▸). It is also in agreement with recent developments made in thermodynamic modelling (*e.g.* Myers *et al.*, 2014 ▸; Richardson, 2008 ▸; Walker *et al.*, 2007 ▸). A sketch of the C–S–H structure under the tobermorite-like assumption is shown in Fig. 1 ▸.

As indicated above, the ambiguities that remain as to which structure model most accurately describes C–S–H evolution as a function of its Ca/Si ratio mainly result from the intense structural disorder reigning in this phase, with additional complexity arising from nanocrystallinity. Disorder occurs first in the arrangement between C–S–H particles (Etzold *et al.*, 2014 ▸; Feldman & Sereda, 1968 ▸) but also and more importantly within the C–S–H crystals themselves. As a consequence of this disorder, X-ray diffraction patterns exhibit a few broad and, for some of them, asymmetric maxima. As this cannot be straightforwardly taken into account in the Rietveld X-ray diffraction (XRD) refinement method, it led to the use of alternative methods which probe the local or medium-range order in the C–S–H structure (*e.g.* ^29^Si NMR, infrared and Raman spectrometries, *etc*.; Cong & Kirkpatrick, 1996*a* ▸,*b* ▸; Kirkpatrick *et al.*, 1997 ▸; Lequeux *et al.*, 1999 ▸; Yu *et al.*, 1999 ▸). Amongst them, ^29^Si NMR was proven particularly efficient and helped to demonstrate that the length of the Si chains decreases with increasing Ca/Si ratio (*e.g.* Brunet *et al.*, 2004 ▸; Cong & Kirkpatrick, 1996*b* ▸). However, this method only provides a partial picture of the structure probed, which induces difficulties of analysis. For example, it may be difficult to distinguish between the Si local environment of jennite-like and 14 Å tobermorite-like structures (Cong & Kirkpatrick, 1996*a* ▸). To circumvent such ambiguities, transmission electron microscopy (TEM) has often been used as an alternative method of analysis, as chemical and morphological data can successfully be retrieved at the crystal scale (Groves *et al.*, 1986 ▸; Richardson *et al.*, 1994 ▸; Richardson & Groves, 1992*b* ▸, 1993 ▸). TEM is also sometimes used to extract structural information by acquiring electron diffraction patterns and interference fringes, which are compared with those of tobermorite and jennite (Viehland *et al.*, 1996 ▸; Zhang *et al.*, 2000 ▸). However, in the case of nanocrystalline and lamellar structures, numerous structural particularities, such as internal strains, bending, aggregation or limited stability under the beam, induce many modulations of the diffraction pattern and may lead to incorrect data interpretation (Chatterji, 1997 ▸).

Recently, it has been proposed that C–S–H X-ray diffraction patterns could be reproduced by considering that C–S–H has a tobermorite-like structure affected by turbostratism (the systematic presence, between adjacent layers that remain parallel, of a random rotation and/or a random translation; Grangeon, Claret, Linard & Chiaberge, 2013 ▸). In this model, most of the diffraction maxima are *hk* bands, whose position, relative intensity and breadth can be exploited to retrieve quantitative data on, for example, the layer structure. Complementary information can be obtained from the study of the 001 reflection [using the indexing proposed by Grangeon, Claret, Lerouge *et al.* (2013 ▸)]. It has been regularly shown that this reflection shifts towards low *d* spacing with increasing Ca/Si ratio (Garbev, Beuchle *et al.*, 2008 ▸; Garbev, Bornefeld *et al.*, 2008 ▸; Grangeon, Claret, Linard & Chiaberge, 2013 ▸; Matsuyama & Young, 2000 ▸; Renaudin *et al.*, 2009 ▸; Richardson, 2014 ▸; Walker *et al.*, 2007 ▸). This may result from different phenomena, including (i) a change in the layer-to-layer distance, (ii) a change in crystallite size along c* (*i.e*. a change in the mean number of layers stacked parallel to each other), and (iii) interstratification, possibly following a random (R0) junction type (Drits & Tchoubar, 1990 ▸; Heller & Taylor, 1956 ▸; Taylor & Howison, 1956 ▸), of different types of layers, including different tobermorite-like layers and (or) Ca(OH)_2_ layers (Brunauer & Greenberg, 1960 ▸; Girão *et al.*, 2010 ▸; Grangeon, Claret, Linard & Chiaberge, 2013 ▸; Heller & Taylor, 1956 ▸; Taylor & Howison, 1956 ▸).

The present study aims to contribute to a better understanding of C–S–H structure. Synchrotron X-ray absorption near-edge structure spectroscopy (XANES) was used to identify the most relevant crystalline analogues of C–S–H and to check for the presence of accessory phases, Fourier-transform infrared (FTIR) spectroscopy to check for the presence of (CO_3_)^2−^ groups that would be indicative of alteration of the samples by atmospheric CO_2_, ^29^Si NMR to probe the connectivity of Si atoms, and powder X-ray diffraction (XRD) to probe crystallite sizes, to obtain the layer-to-layer distance and to cross-check the ^29^Si NMR results. All of the results are gathered to propose a model for the structural evolution of C–S–H as a function of its Ca/Si ratio.

## 2. Materials and methods   

### 2.1. Samples   

C–S–H samples were synthesized by mixing different molar ratios of Ca(OH)_2_ (Kanto Chemical, special grade) and amorphous SiO_2_ (Aerosil200) in a glove-box (under N_2_ atmosphere) and in CO_2_-free de-ionized water (Table 1 ▸). The target atomic Ca/Si ratios were 0.6, 0.83, 1.0, 1.4 and 1.5. The effect of temperature was investigated by performing syntheses at room temperature (RT), 323 K, 353 K and 443 K (using an autoclave). All suspensions were stirred during synthesis. Samples are labelled CSH *X*–*Y* K, where *X* is the target Ca/Si ratio and *Y* the synthesis temperature. The chemical composition of all samples is reported in Table 1 ▸. The same protocol, but at a temperature of 453 K and a pressure of 10^3^ Pa, was applied for synthesizing tobermorite. This synthetic tobermorite, whose Ca/Si ratio is 0.82 ± 0.02, has a lath-like shape with typical dimensions of 1–2 × 0.1–0.5 µm (see supporting information).

After synthesis, all samples were filtrated, freeze-dried and finally left in closed containers in the glove-box until analysis. Note that, despite all precautions taken, samples may have been in contact with minor amounts of water present in the glove-box atmosphere, which may have a minor influence on their structure (adsorption of water on external and interlayer surfaces; *e.g.* Korpa & Trettin, 2006 ▸; Taylor, 1986 ▸; Taylor & Howison, 1956 ▸).

In addition to these synthetic products, synthetic calcite (CaCO_3_) and portlandite [Ca(OH)_2_], as well as natural dolomite (Ca_0.5_Mg_0.5_CO_3_ from Brumado, Brazil), ettringite [Ca_6_Al_2_(SO_4_)_3_(OH)_12_·26H_2_O; N’Chwaning, South Africa] and gypsum (CaSO_4_·2H_2_O; Miyazaki, Japan), were selected to serve as references.

### 2.2. Electron probe micro-analysis   

Electron probe micro-analysis (EPMA) of natural and synthetic C–S–H was performed on polished thin sections, made from pressed sample pellets, using a Cameca SX50 electron microprobe (acceleration voltage of 15 kV, current beam of 12 nA) and a 1–2 µm beam width. Prior to analysis, a 10–20 nm-thick carbon layer was sputter-coated onto the samples (Edwards Auto 306). Ca and Si were analysed simultaneously. Ca *K*α and Si *K*α were analysed using a pentaerythritol crystal and a thallium acid phthalate crystal, respectively. The standards used were albite (NaAlSi_3_O_8_) for Si and wollastonite (CaSiO_3_) for Ca. A ZAF data correction was applied to the raw data.

### 2.3. Synchrotron X-ray absorption near-edge structure spectroscopy   

Ca *K*-edge absorption spectra were measured at beamlines 9A and 12C of the Photon Factory, KEK, Tsukuba, Japan (Nomura & Koyama, 2001 ▸). Spectra were recorded in fluorescence mode using a Lytle type detector. Synchrotron radiation from the 2.5 GeV storage ring was monochromated with Si(111) crystals. The incident beam was collimated to 1 × 1 mm. The energy was calibrated by using a Cu foil and calibrating the pre-edge peak at 8980 eV. Data reduction was performed following previous studies (Isaure *et al.*, 2002 ▸) and using software from the Advanced Light Source (Berkeley, USA) 10.3.2 beamline (Marcus *et al.*, 2004 ▸). Samples were protected from the atmosphere during measurement by using tape.

### 2.4. Fourier-transform infrared spectrometry   

FTIR spectra were obtained on a JASCO-FT/IR-4100 spectrometer. For each sample, 1 mg of powder was mixed with 100 mg of KBr and pressed to produce a pellet. Thirty-two transmission scans were performed in the 4000–350 cm^−1^ spectral range with a resolution of 0.024 cm^−1^ and averaged for each spectrum. The spectrum of CSH 0.6–323 K could only be recorded in the 3000–350 cm^−1^ range.

### 2.5. ^29^Si magic angle spinning (MAS) nuclear magnetic resonance   

^29^Si NMR spectra were recorded on a Bruker AVANCE 7.4 T operated at 59 MHz and equipped with a 4 mm double bearing MAS probe head spinning at 12 kHz. About 16 000 scans were accumulated after a 45° pulse, using a 10 s recycling delay. This delay was optimized to ensure a complete relaxation of the magnetization. ^29^Si chemical shifts were reported relative to tetramethylsilane resonance. The spectra were simulated as a sum of individual Gaussian–Lorentzian functions, using the *Dmfit* program (Massiot *et al.*, 2002 ▸). Their integrated intensities were used to estimate the amount of the differently coordinated species. The mean Si chain length (*i.e*. the mean number of Si atoms that are connected in a chain) was calculated following Richardson (2014 ▸).

### 2.6. Powder X-ray diffraction   

XRD was performed with a Rigaku RINT-2000, operated at 30 kV and 20 mA, using Cu *K*α radiation (λ = 1.5418 Å), a divergence slit of 1°, a scatter slit of 1° and a receiving slit of 3 mm. Intensities were recorded in continuous mode, at a scan rate of 1° min^−1^, and were integrated every 0.05° 2θ. Simulations of *hk* bands were performed using software adapted to the study of defective lamellar structures (Plançon, 2002 ▸). This software is based on a matrix formalism (Drits & Tchoubar, 1990 ▸), briefly overviewed in a previous publication (Grangeon, Claret, Linard & Chiaberge, 2013 ▸), which was previously successfully used for the analysis of C–S–H structure and alteration mechanisms (Grangeon, Claret, Lerouge *et al.*, 2013 ▸; Grangeon, Claret, Linard & Chiaberge, 2013 ▸; Marty *et al.*, 2015 ▸), as well as for the analysis of the structure of other cement phases (Roosz *et al.*, 2015 ▸) and of nanocrystalline and defective manganese (*e.g.* Grangeon *et al.*, 2008 ▸, 2010 ▸) and iron (Hadi *et al.*, 2014 ▸) oxides. For all calculations, the structure model of an 11 Å tobermorite (Merlino *et al.*, 2001 ▸) was adapted, and the coherent scattering domain size in the *ab* plane was set to 10 nm. Owing to sample turbostratism, **c** cannot be defined (see *e.g.* Bish & Post, 1990 ▸; Brindley & Brown, 1980 ▸; Drits & Tchoubar, 1990 ▸), and c* (perpendicular to the *ab* plane) will be used instead to refer to the direction perpendicular to the *ab* plane (layer plane). Evolution of the intensity diffracted at ∼16.1° 2θ Cu *K*α as a function of sample Ca/Si ratio was monitored using the following formula: *I*_rel_ = [(*I*_16.1°_*i*_/*I*_29.2°_*i*_)_Ca/Si_*i*_]/[(*I*_16.1°_0.6_/*I*_29.2°_0.6_)_0.6_]. In this calculation, (*I*_16.1°_*i*_/*I*_29.2°_*i*_)_Ca/Si_*i*_ stands for the intensity (background subtracted) of the maximum of the band at ∼16.1° 2θ Cu *K*α relative to the maximum of the band at ∼29.2° 2θ Cu *K*α, for a given sample, and (*I*_16.1°_0.6_/*I*_29.2°_0.6_)_0.6_ is the same calculation, but made for the sample of lowest Ca/Si ratio (here, about 0.6). Consequently, the evolution of *I*_rel_ as a function of Ca/Si depicts the evolution of the intensity diffracted at ∼16.1° 2θ Cu *K*α relative to the maximum at ∼29.2° 2θ Cu *K*α and to the sample of lowest Ca/Si.

The formalism used for the modelling of 00*l* reflections (Plançon, 2002 ▸) allows for a change in the layer-to-layer distance without affecting the absolute coordinates (in ångströms) of atoms along the normal to the layer (coordinates along **a** and **b** are not needed for the calculation of 00*l* reflections). In other words, all distances and angles between layer atoms are kept identical to those of the original structure model [in this case, the model from Merlino *et al.* (2001 ▸)], while the interlayer spacing is varied. For a detailed description of the mathematical formalism used for the modelling of 00*l* reflections, the reader is referred to previous publications (*e.g.* Drits *et al.*, 1993 ▸; Plançon, 1981 ▸; Moore & Reynolds, 1989 ▸; Sakharov & Lanson, 2013 ▸). During the refinement of the 001 reflection, the sole free parameters were the layer-to-layer distance and the crystallite size perpendicular to the layer plane; that is, the mean number of layers stacked coherently (*i.e.* parallel to each other, albeit subject to random translation or rotations in the *ab* plane). The occupancies of Si bridging tetrahedra and of interlayer Ca were, respectively, constrained using ^29^Si NMR and EPMA. All instrumental parameters were constrained from the geometry of the experiment. The background was constrained to be the same for all samples and to be linearly decreasing with increasing ° 2θ values. Fit quality was evaluated with the usual *R*_wp_, *R*_exp_ and goodness of fit (GoF) factors (Howard & Preston, 1989 ▸). Although the C–S–H structure is suspected to be subject to interstratification, this phenomenon was not considered here, because only one of the 00*l* reflections was observed, which does not provide enough information to constrain a possible interstratification phenomenon, and because this reflection was approximately symmetrical. Note that, in the samples of highest Ca/Si ratio, the presence of nanocrystalline Ca(OH)_2_ sandwiched between two C–S–H layers (see below) could be understood as an interstratified structure, but can be described using a unique unit cell, because of the regular and systematic alternation, along c*, of the two types of ‘layers’.

## 3. Results   

### 3.1. X-ray absorption   

**Figure 2:**
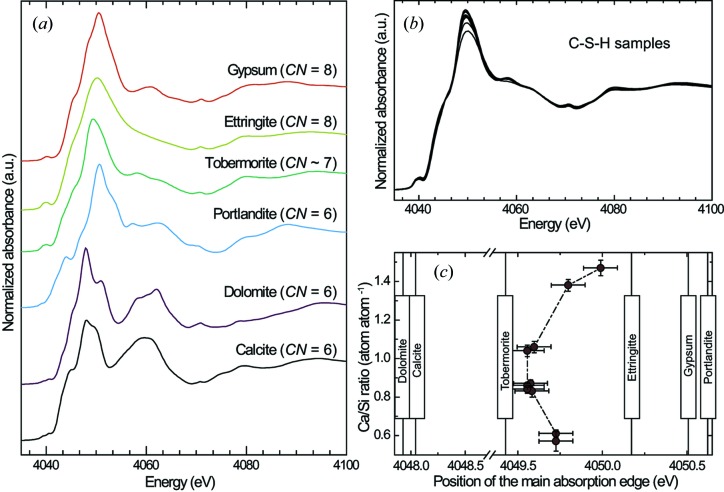
Reference (*a*) and C–S–H (*b*) XANES spectra. *CN* stands for ‘calcium coordination number’. See text for details. (*c*) The relation between Ca/Si ratio and position of the main edge (dots) in each of the studied C–S–H samples as well as the position of the main edge in each of the references (vertical lines with associated caption).

XANES spectra of all samples and reference compounds are presented in Fig. 2 ▸. In all of the references, Ca is in the Ca^2+^ oxidation state, and the position of the main adsorption edge varies between 4047.9 eV (dolomite) and 4050.6 eV (portandite). In these references, the number of oxygen atoms to which Ca is bound is six (calcite, dolomite and portlandite), seven (tobermorite), eight (ettringite) or nine (gypsum). In agreement with literature data (Sowrey *et al.*, 2004 ▸), the position of the main edge generally shifts towards higher energy when Ca coordination number increases, with the noticeable exception of portlandite, which has both the smallest Ca coordination number (six) and the highest absorption edge energy (4050.6 eV). These observations are in agreement with literature data (Michel *et al.*, 2008 ▸), in which the difference in the position of the main absorption edge of calcite and portlandite was found to be +2.8 eV (+2.6 eV in the present study) and the difference between the calcite and aragonite edges +1.3 eV (+1.5 eV in the present study). Finally, it should be noted that Ca was assumed to be sevenfold coordinated in tobermorite (*i.e.* only layer Ca was considered), although the coordination number may actually be slightly lower, owing to the possible presence of interlayer Ca whose coordination number may vary, for example as a function of sample hydration.

A first comparison of all C–S–H spectra (Fig. 2 ▸ *b*) reveals no obvious difference between them, there being a generally similar Ca local environment over the whole range of Ca/Si ratio investigated. Amongst all the references, the tobermorite spectrum is the closest to the C–S–H spectra, which shows that Ca has a similar local environment in tobermorite and all C–S–H samples studied here. A closer examination, using derivative spectra (Fig. 2 ▸ *c*), reveals that the main absorption edge of samples having a target Ca/Si ratio of 0.83 (Table 1 ▸) is at 4049.6 ± 0.1 eV, close to the tobermorite spectrum whose main edge is at 4049.4 eV. This indicates a close structural similarity between these C–S–H samples and tobermorite. Within uncertainties, the main edge is at the same position in these four samples and in the samples having a target Ca/Si ratio of 0.6 and of 1.0. Consequently, the Ca local environment in these samples is, within the uncertainties, identical. Contrastingly, and relative to the samples having a target Ca/Si ratio of 0.83, the position of the main edge shifts towards higher energy when the Ca/Si ratio increases, up to 4050.0 eV for CSH 1.5–443 K. This might reflect a change in Ca local environment when the structure slightly deviates from that of tobermorite but also the presence of Ca-rich impurities in samples having the highest Ca/Si ratios (CSH 1.4–323 K and CSH 1.5–443 K). From analysis of literature data, this impurity could be Ca(OH)_2_ (*e.g.* Richardson, 2004 ▸, 2008 ▸, 2014 ▸; Richardson & Groves, 1992*a* ▸). This would be compatible with the present results, as the main edge of portlandite is at 4050.6 eV. Note that the presence of ettringite and (or) gypsum is not expected in the present samples, as the parent solution used for synthesis did not contain either Al or S, and as these two elements could not be detected by EPMA.

### 3.2. Fourier-transform infrared spectrometry   

**Figure 3:**
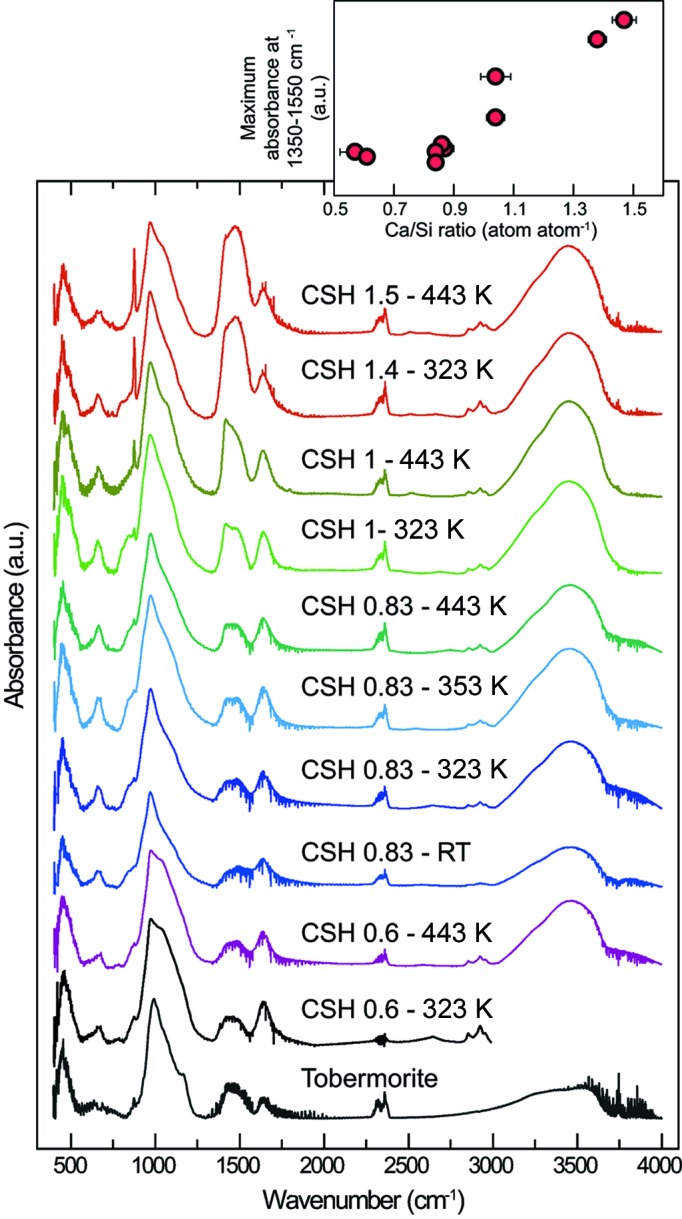
Main panel: FTIR spectra of all studied C–S–H samples. From top to bottom, spectra are sorted by decreasing target Ca/Si ratio and by decreasing synthesis temperature. The inset at the top right shows the evolution of the maximum of the absorbance at 1350–1550 cm^−1^ as a function of sample Ca/Si ratio.

The FTIR spectra of all of the C–S–H samples (Fig. 3 ▸) are very close to the tobermorite spectrum (Fig. 3 ▸; Yu *et al.*, 1999 ▸) but differ from the jennite spectrum which has numerous absorption maxima, for example between 3465 and 3740 cm^−1^ (Carpenter *et al.*, 1966 ▸; Yu *et al.*, 1999 ▸).

In all of the spectra [which are here interpreted following Yu *et al.* (1999 ▸)], the series of bands in the 400–500 cm^−1^ range is related to Si—O linkages, and the band at about 650 cm^−1^ is related to Si—O—Si bending vibrations, influenced by the Si—O—Si angle and the occupancy of neighbouring sites. The main band at 970 cm^−1^ is assigned to asymmetric stretching vibrations of Si—O generated by *Q* ^2^ units (see §3.3 for the definition of *Q* ^2^). The band at about 1630 cm^−1^ is ascribed to H—O—H bending vibrations of molecular H_2_O, and the broad band centred around 3430 cm^−1^ to O—H stretching vibrations. Such assignments were confirmed using *ab initio* molecular dynamics (Churakov, 2009*a* ▸,*b* ▸). The maxima appearing in the range 1350–1550 cm^−1^ are attributed to (CO_3_)^2−^. The presence of a peak at 875 cm^−1^ suggests this is calcium carbonate. The evolution of the intensity of the bands at 875 and 1350–1550 cm^−1^ as a function of Ca/Si ratio is best described with a two-step process (inset in Fig. 3 ▸): the absorbance is low and constant when the Ca/Si ratio is lower than or equal to 0.87 ± 0.02 and then increases with the Ca/Si ratio at higher ratios. As samples were kept in an N_2_-saturated glove-box, and were exposed to air only a few tens of minutes prior to measurement, the sensitivity to fast carbonation processes increases with the Ca/Si ratio. In agreement with the XANES results, this is best explained by assuming that samples having a Ca/Si ratio higher than 0.87 ± 0.02 contain a growing proportion of a Ca-rich impurity that would be sensitive to carbonation, such as Ca(OH)_2_. Similar preferential carbonation of samples of high Ca/Si ratio was observed in a series of eight dried samples having Ca/Si ratios ranging between 0.41 and 1.70 (Yu *et al.*, 1999 ▸).

### 3.3. ^29^Si nuclear magnetic resonance   

**Figure 4:**
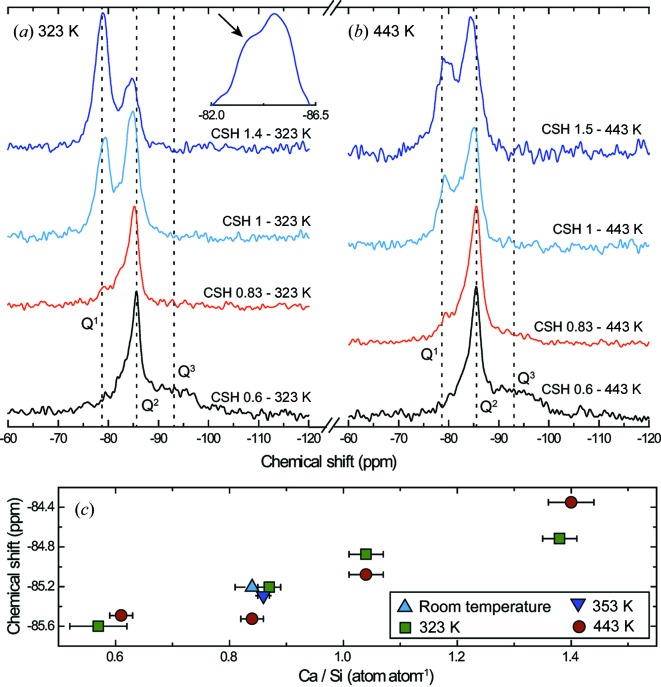
^29^Si MAS NMR spectra acquired on C–S–H samples synthesized at 323 K (*a*) and 443 K (*b*), and evolution of the *Q* ^2^ chemical shift as a function of sample Ca/Si ratio (*c*). In (*a*) and (*b*), spectra are sorted, from top to bottom, by decreasing Ca/Si ratio and dashed vertical lines show the approximate position of, from left to right, Si *Q* ^1^, *Q* ^2^ and *Q* ^3^ environments. The inset in (*a*) points, with the example of CSH 1.4–323 K, to the appearance of a shoulder on the resonance of the *Q* ^2^ environment when the Ca/Si ratio increases. In (*c*), samples synthesized at room temperature, 353 K, 323 K and 443 K are, respectively, shown as up and down triangles, squares and dots.

C–S–H ^29^Si MAS NMR spectra are shown in Fig. 4 ▸. The three main resonances at −79/−80, −84.5/−85.5 and −92/−94 ppm are respectively assigned, following previous literature studies (Brunet *et al.*, 2004 ▸; Cong & Kirkpatrick, 1996*b* ▸; Cong & Kirkpatrick, 1996*a* ▸; Maeshima *et al.*, 2003 ▸; Pardal *et al.*, 2012 ▸; Richardson *et al.*, 2010 ▸), to *Q* ^1^, *Q* ^2^ and *Q* ^3^ sites. These three Si sites are typical for calcium silicate hydrates (Fig. 1 ▸). In a *Q* ^1^ site, an Si atom is only connected to another Si atom, and this site is generally assigned to Si atoms forming paired tetrahedra at the surface of the C–S–H Ca layer (Fig. 1 ▸). In a *Q* ^2^ site, an Si atom is connected to two other Si atoms, and this site corresponds either to an Si atom bridging two of the aforementioned Si atoms or to an Si atom from paired tetrahedra connected to this bridging Si tetrahedron. As previously observed (*e.g.* Lequeux *et al.*, 1999 ▸; Noma *et al.*, 1998 ▸), the chemical shift associated with the *Q* ^2^ environment varies with the Ca/Si ratio (Fig. 4 ▸ *c*), from −85.60 ppm (CSH 0.6–323 K) to −84.35 ppm (CSH 1.5–443 K). This is probably linked to an evolution of Si local environment, as discussed below. Finally, the nature of the *Q* ^3^ site is subject to discussion, in particular because the corresponding band is broad (Fig. 4 ▸). It may be assigned to a bridging Si atom connected to another one from the adjacent layer through its apical oxygen (Fig. 1 ▸; Trapote-Barreira *et al.*, 2014 ▸), or to silanols resulting from incomplete dissolution of the amorphous silica used for synthesis (*e.g.* Brinker *et al.*, 1988 ▸; Leonardelli *et al.*, 1992 ▸). The presence of silanols, which are remnants from the synthesis reactants, is likely in CSH 0.6–323 K and CSH 0.6–443 K, as their Ca/Si ratio is lower than the minimum value of 2/3 that can be obtained assuming a tobermorite-like structure. Taking into account this chemical constraint and assuming that these samples contain a mix of silanols and of a C–S–H having a Ca/Si ratio of 2/3, CSH 0.6–323 K and CSH 0.6−443 K, respectively, contain 7–22 and 0–12% of silanols.

**Figure 5:**
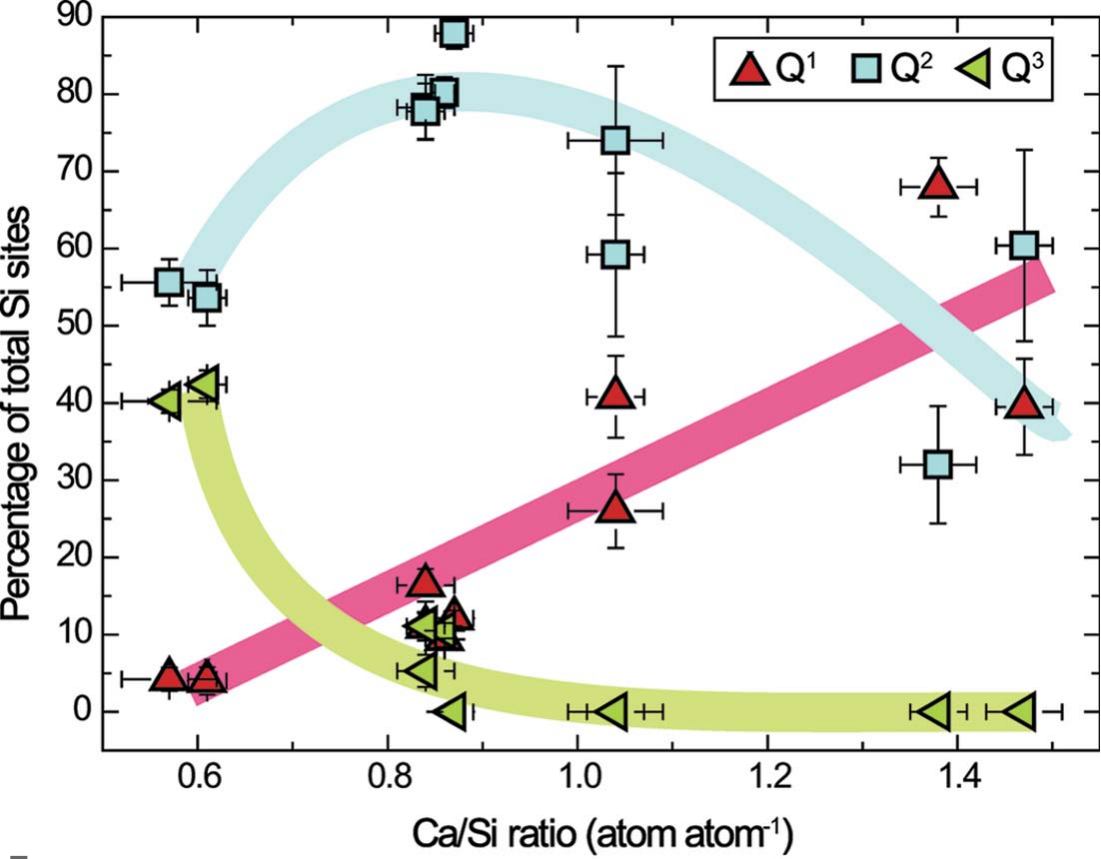
Evolution of the proportion of Si *Q* ^1^ (triangles pointing to the top), *Q* ^2^ (squares) and *Q* ^3^ (triangles pointing to the left) environments as a function of sample Ca/Si ratio, as retrieved from analysis of ^29^Si MAS NMR data. Solid lines are intended to be guides for the eye.

The proportion of each Si environment does not depend on synthesis temperature, whereas the Ca/Si ratio has a major influence (Table 2 ▸ and Fig. 5 ▸). The proportion of *Q* ^1^ environment increases with the Ca/Si ratio, from 4.2 ± 1.5% and 4.0 ± 1.8% for, respectively, CSH 0.6–323 K (Ca/Si ratio of 0.57 ± 0.05) and CSH 0.6–443 K (Ca/Si ratio of 0.61 ± 0.02) up to 68.1 ± 3.8% for CSH 1.4–323 K (Ca/Si ratio of 1.38 ± 0.03). The evolution of the *Q* ^2^ environment is more complex, being equal to 55.6 ± 3.0% and 53.6 ± 3.6% for, respectively, CSH 0.6–323 K and CSH 0.6–443 K, increasing up to 87.9 ± 2% for CSH 0.83–323 K (Ca/Si ratio of 0.87 ± 0.02) and then decreasing at higher Ca/Si ratios, down to 32.0 ± 7.6% for CSH 1.4–323 K (Ca/Si ratio of 1.38 ± 0.03). Finally, the proportion of *Q* ^3^ environment decreases with increasing Ca/Si ratio, from 40.2 ± 1.5% (CSH 0.6–323 K) down to 0 when the Ca/Si ratio is 0.87 ± 0.02 or higher. From chemical considerations (see above), even if silanols could be present in CSH 0.6–323 K and CSH 0.6–443 K, they do not account for more than 12% of the total Si in CSH 0.6–443 K and 22% in CSH 0.6–323 K. Thus, these samples contain Si in a *Q* ^3^ configuration. For the three other samples in which a *Q* ^3^ environment is detected (CSH 0.83–RT, CSH 0.83–353 K and CSH 0.83–443 K), the ratio of *Q* ^3^ to *Q* ^1^ is lower than that expected for a double-chain tobermorite [*Q* ^3^ = 1/3 − *Q* ^1^/2 following Richardson (2014 ▸)], probably because of the nanocrystallinity of the presently studied samples. Indeed, with 3–4 layers stacked coherently on average (see below), 25–33% of Si wollastonite-like chains are exposed at the particle surface, in which all Si bridging tetrahedra are connected at most to two Si atoms (*Q* ^2^ environment) instead of three (*Q* ^3^ environment) in the interlayer space.

The main length of Si chain (the mean number of Si tetrahedra connected in the wollastonite-like chains) decreases with the Ca/Si ratio (Table 2 ▸), being equal to 48 for the lowest Ca/Si ratio (0.57 ± 0.05) and having a minimum value of 3 (Ca/Si = 1.38 ± 0.03).

### 3.4. Powder X-ray diffraction   

**Figure 6:**
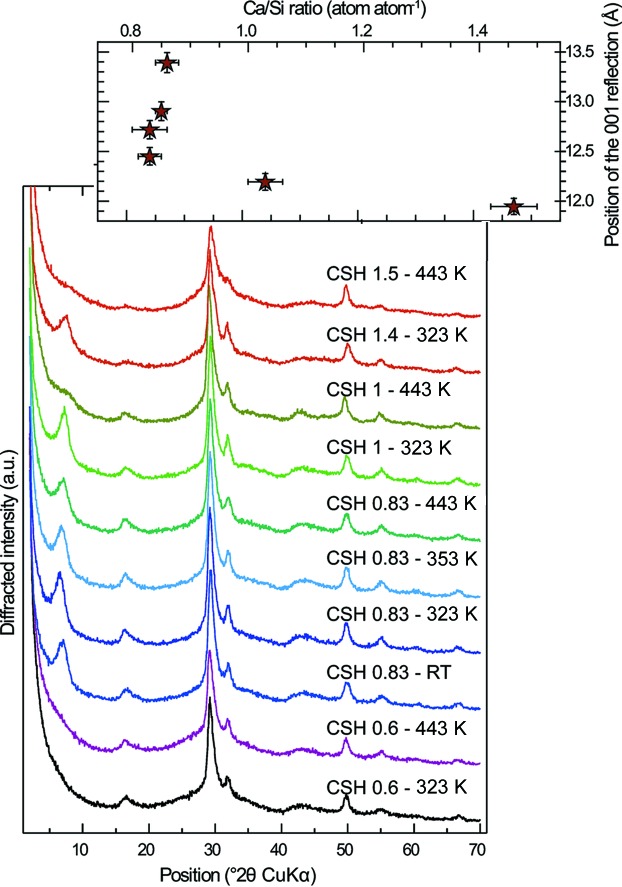
Main panel: XRD pattern of all studied samples, sorted as in Fig. 3 ▸. The inset at the top right shows the evolution of the position of the 001 reflection (when observable) as a function of sample Ca/Si ratio.

The XRD patterns of all of the C–S–H samples have a high degree of similarity (Fig. 6 ▸). They are all attributable to a tobermorite-like structure affected by nanocrystallinity and turbostratism (Grangeon, Claret, Lerouge *et al.*, 2013 ▸; Grangeon, Claret, Linard & Chiaberge, 2013 ▸), the former inducing broad diffraction maxima and the latter cancelling *hkl* reflections with *h* ≠ 0 and *k* ≠ 0 (Drits & Tchoubar, 1990 ▸). All maxima are 00*l* reflections and *hk* bands.

**Figure 7:**
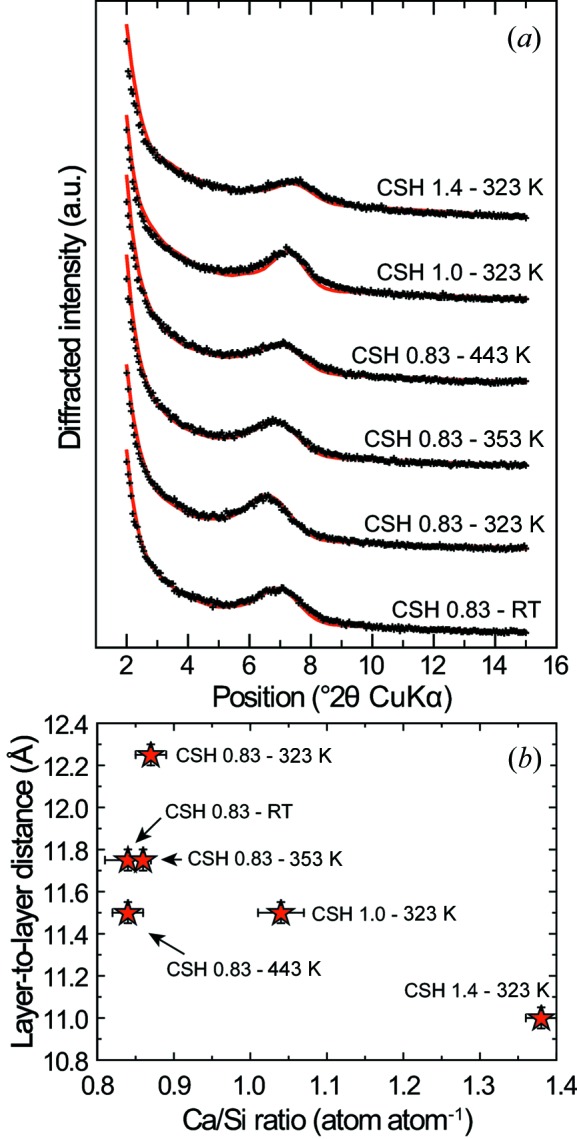
(*a*) Data (black crosses) and best simulation (red solid line) of the 001 reflection from, from top to bottom, CSH 1.4–323 K, CSH 1.0–323 K, CSH 0.83–443 K, CSH 0.83–353 K, CSH 0.83–323 K and CSH 0.83–RT XRD patterns. The deduced evolution of the layer-to-layer distance as a function of sample Ca/Si ratio is shown in (*b*). All simulation results are given in Table 3 ▸.

Amongst the diffraction maxima, the 001 reflection (at ∼7.4° 2θ Cu *K*α) has the most pronounced variations, both in position and in intensity (Fig. 6 ▸). It is absent in three samples (CSH 0.6–323 K, CSH 0.6–443 K and CSH 1.5–443 K) and very weak in a fourth sample (CSH 1.0–443 K). In these samples, crystallites are thus overwhelmingly built of isolated layers, which means that the crystals are essentially made of isolated layers or of layers not stacked parallel to each other. When observable, the 001 reflection shifts towards low *d* spacing (high diffraction angles) with increasing Ca/Si ratio (inset in Fig. 6 ▸), from 12.9 ± 0.4 Å for samples having a target Ca/Si ratio of 0.83, down to 11.9 ± 0.1 Å for the sample having a Ca/Si ratio of 1.38 ± 0.03. Such variation is commonly observed (Garbev, Beuchle *et al.*, 2008 ▸; Matsuyama & Young, 2000 ▸; Renaudin *et al.*, 2009 ▸; Richardson, 2014 ▸; Walker *et al.*, 2007 ▸) and cannot be related, in the present study, to a change in the mean number of layers stacked parallel to each other with varying Ca/Si ratio, as the full width at half-maximum of this reflection is similar in all samples. The evolution of the layer-to-layer distance was further assessed by modelling the 001 reflection when it was present (Fig. 7 ▸ and Table 3 ▸). Consistently with qualitative observation, the layer-to-layer distance decreases from 12.25 to 11.50 Å for samples having a Ca/Si ratio varying between 0.84 ± 0.02 and 0.87 ± 0.02, down to 11 Å for CSH 1.4–323 K which has a Ca/Si ratio of 1.38 ± 0.03. Such values are about 1 Å smaller than those deduced from the qualitative examination of the position of the 001 reflection, as a result of sample nanocrystallinity (Drits & Tchoubar, 1990 ▸; Reynolds, 1968 ▸, 1986 ▸). This demonstrates that any study of the layer-to-layer distance in nanocrystalline layered phases cannot rely solely on the qualitative study of this reflection. The nanocrystallinity of the presently studied samples is confirmed by the results of these calculations, as the crystallite size perpendicular to the layer plane is 4–5 nm for all modelled patterns (Table 3 ▸).

**Figure 8:**
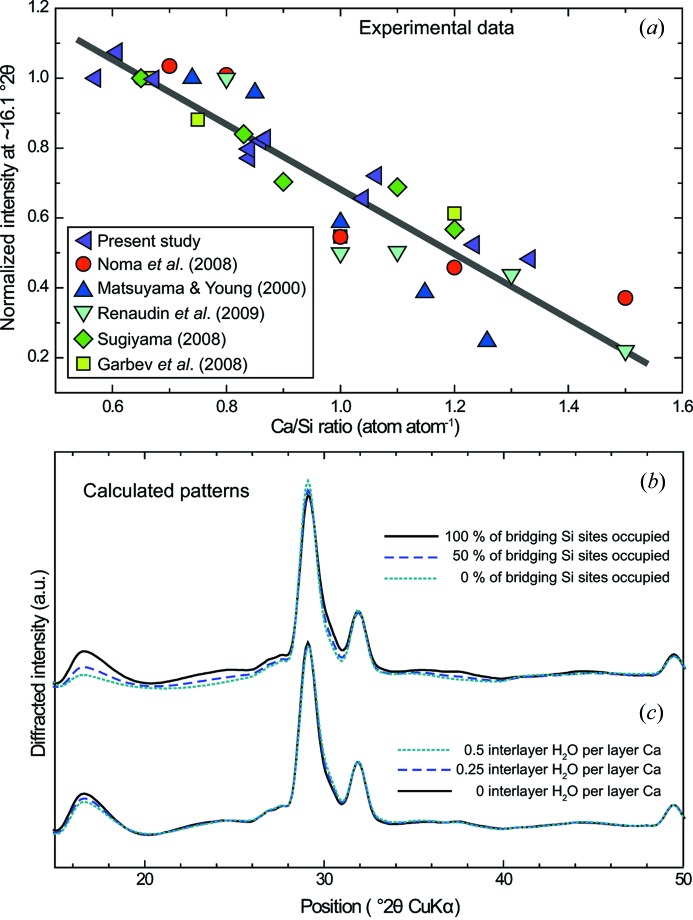
(*a*) Evolution of the normalized intensity of the diffraction maximum at ∼16.1° 2θ Cu *K*α (*I*_rel_; see text for details) as a function of sample Ca/Si ratio. Data presented in Fig. 4 ▸ and additional data acquired for the present study (XRD patterns not shown) are shown with solid purple triangles pointing to the left. These data are compared with literature data, shown with solid red circles (Noma *et al.*, 1998 ▸), solid blue triangles pointing to the top (Matsuyama & Young, 2000 ▸), solid cyan triangles pointing to the bottom (Renaudin *et al.*, 2009 ▸), solid green diamonds (Sugiyama, 2008 ▸) and solid light-green squares (Garbev, Beuchle *et al.*, 2008 ▸; Garbev, Bornefeld *et al.*, 2008 ▸). Only studies performed on dried samples were considered, as this band may be influenced by the content of interlayer water. Data scattering is probably a result of the normalization procedure (related both to the data digitalization method and to the fact that some studies did not present data for samples having a Ca/Si of ∼0.6) and a possible variable content of, for example, interlayer Ca. The solid grey line is the best linear fit to the data (*y* = −0.913*x* + 1.597; *r* ^2^ = 0.9). (*b*), (*c*) Calculations that show the dependence of XRD patterns on the occupancy of Si bridging tetrahedra in the wollastonite-like chains (*b*) (solid black, dashed purple and dotted blue lines are calculations where the occupancy of Si bridging tetrahedra is, respectively, 1, 0.5 and 0) and on the abundance of interlayer water (*c*) (solid black, dashed purple and dotted blue lines are calculations where the number of interlayer water molecules is, respectively, 0.5, 0.25 and 0 per layer calcium).

Another variation in intensity is observed on the band at ∼16.1° 2θ Cu *K*α (Fig. 6 ▸). When normalized to the intensity of the band at ∼29.2° 2θ Cu *K*α, it steadily decreases in intensity with increasing Ca/Si ratio, in agreement with previous observations (Fig. 8 ▸). From analysis of literature data and the present study, the main parameters that are susceptible to evolving as a function of the Ca/Si ratio are the occupancy of Si bridging tetrahedra in the wollastonite-like chains (*e.g.* Myers *et al.*, 2013 ▸; Richardson, 2014 ▸) and the abundance of interlayer water (Kim *et al.*, 2013 ▸; Marty *et al.*, 2015 ▸). The impact of these two parameters on XRD patterns was tested here (Fig. 8 ▸). It can be observed that the intensity of the band at ∼16.1° 2θ Cu *K*α mainly depends on the occupancy of Si atoms in the bridging site, with the diffracted intensity decreasing with decreasing Si occupancy (Fig. 8 ▸ *b*). The abundance of interlayer water has a minor effect (Fig. 8 ▸ *c*). Consequently, XRD and ^29^Si NMR consistently show that as the Ca/Si ratio increases the wollastonite-like chains depolymerize through omission of bridging Si tetrahedra. This is in agreement with the study of Matsuyama & Young (2000 ▸), who also observed, using ^29^Si NMR, that Si chains depolymerize when the sample Ca/Si ratio increases, and who provided XRD patterns in which it can be observed that the intensity of the band at ∼16.1° 2θ Cu *K*α relative to that of the band at ∼29.2° 2θ Cu *K*α weakens when the Ca/Si ratio increases (Fig. 8 ▸ *a*).

## 4. Discussion   

### 4.1. Evolution of C–S–H structure as a function of its Ca/Si ratio   

The present study provides evidence for C–S–H being nanocrystalline and turbostratic tobermorite over the whole range of Ca/Si ratio investigated. A mechanism for the structure evolution as a function of Ca/Si ratio is now proposed in the following sections.

#### 4.1.1. Structure of the samples of low Ca/Si ratio (CSH 0.6–323 K and CSH 0.6–443 K)   

In CSH 0.6–323 K and CSH 0.6–443 K (Ca/Si ratios of 0.57 ± 0.05 and 0.61 ± 0.02), the *Q* ^3^ environment accounts for 40–42% of the total Si and, even if part of these sites are remnants from the reactants used for the synthesis (see above), layer-to-layer connectivity through bridging Si tetrahedra exists. In apparent contradiction, XRD shows that crystallites are built of isolated layers. However, because XRD probes crystallites (*i.e.* coherent scattering domains) and not crystals, the apparent discrepancy between XRD and ^29^Si NMR can straightforwardly be reconciled by assuming that stacking disorder occurs not only *via* turbo­stratism but also *via* loss of parallelism between adjacent layers. This may be understood as resulting from a slight corrugation of the layers [as previously schematized (Brunauer & Greenberg, 1960 ▸; Feldman & Sereda, 1968 ▸; Jennings, 2008 ▸) or observed (Marty *et al.*, 2015 ▸; Richardson *et al.*, 2010 ▸)]. As successive layers are connected through Si bridging tetrahedra, random translations (and rotations) in the *ab* plane certainly are of limited amplitude and, by analogy with tobermorite, the layer-to-layer distance in CSH 0.6–323 K and CSH 0.6–443 K is certainly close to 11.3 Å (Merlino *et al.*, 1999 ▸, 2001 ▸). In addition, some of the bridging Si tetrahedra in a *Q* ^3^ configuration may be missing, thus allowing for a local change in the layer-to-layer distance, which would be compatible with layer corrugation. Taking all this information into account, the structure may be close to a defective clino­tobermorite (Richardson, 2014 ▸), being affected by turbo­stratism and layer corrugation. It should be noted that other studies have observed a 001 reflection at about 14 Å in samples of comparable Ca/Si ratio (Grangeon, Claret, Linard & Chiaberge, 2013 ▸; Matsuyama & Young, 2000 ▸; Richardson, 2014 ▸), which could in certain cases indicate that layer-to-layer connectivity through Si bridging tetrahedra is absent (Garbev, Beuchle *et al.*, 2008 ▸; Garbev, Bornefeld *et al.*, 2008 ▸). This means that different C–S–H preparation and (or) conditioning or ageing methods can lead to slightly different C–S–H structures, and the results obtained here for the C–S–H samples having Ca/Si ratios of ∼0.6 are probably not applicable to all C–S–H specimens.

The presence of the Si *Q* ^1^ environment in these samples (Table 2 ▸) may be understood as the presence of isolated paired Si tetrahedra, which would require a locally strong Si chain depolymerization. However, a crystal that is 10 nm in width in the *ab* plane (as reported for samples synthesized using the same method; Grangeon, Claret, Lerouge *et al.*, 2013 ▸) and has defect-free Si chains contains ∼33% *Q* ^3^, ∼63% *Q* ^2^ and ∼5% *Q* ^1^, these latter resulting from border truncation effects. This is in good agreement with experimental values (Table 2 ▸), especially when the contribution of silanols originating from the reactants used for sample synthesis is taken into account, and indicates that the *Q* ^1^ sites detected in CSH 0.6–323 K and CSH 0.6–443 K result mainly from sample nanocrystallinity. The minute size of C–S–H crystals can be further assessed using ^29^Si NMR, as the mean Si chain length (Table 2 ▸) is calculated to be at most (because of the excess of *Q* ^3^) 48–50 Si atoms (∼12 nm).

#### 4.1.2. Structural evolution with increasing Ca/Si ratio   

**Figure 9:**
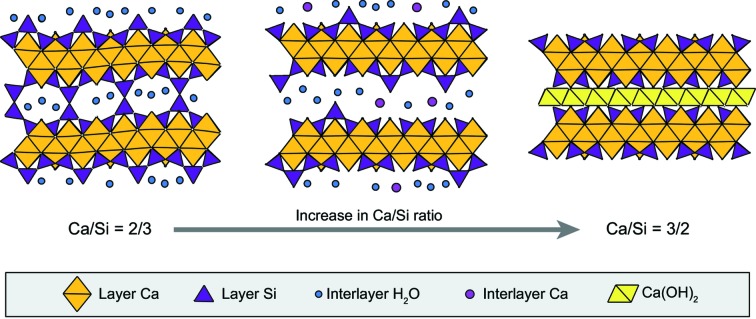
Proposed evolution of C–S–H structure as a function of its Ca/Si ratio. At low Ca/Si ratio, the structure is a nanocrystalline turbostratic tobermorite having corrugated layers. When the Ca/Si ratio increases, Si bridging tetrahedra are progressively removed. A first step of structural evolution consists in an increase of the layer-to-layer distance, because layer-to-layer connectivity weakens. In a second step, layer-to-layer distance decreases, because interlayer Ca is progressively incorporated and holds the layers together. In a final step, Ca coordination spheres connect to form interlayer Ca(OH)_2_ resembling portlandite. The structure of samples having a Ca/Si of ∼1.5 shares a number of similarities with the Richardson (2014 ▸) model, except that that model is three-dimensionally ordered, whereas the present one assumes turbostratism, but these two models can straightforwardly be reconciled (see text). The final step of structural evolution could not be observed here, as all samples contained some Si bridging tetrahedra. Note that the structure of the samples of lowest Ca/Si ratio may vary depending on the synthesis procedure, as some other studies have observed the absence of connectivity between adjacent layers (see text for details).

A scheme of the proposed C–S–H structural evolution as a function of Ca/Si ratio, detailed here below, is proposed in Fig. 9 ▸.

As the Ca/Si ratio increases from 0.57 ± 0.05 to 0.86 ± 0.01, the proportion of Si *Q* ^3^ environment decreases down to 5.3 ± 2.1%, while the proportions of *Q* ^1^ and *Q* ^2^ environments increase and, respectively, peak at 16.4 ± 2.1% and 80.2 ± 2%. Thus, when the Ca/Si ratio increases, depolymerization of the wollastonite-like Si chain occurs, certainly *via* omission of bridging Si tetrahedra as supported by XRD (Fig. 8 ▸), and layer-to-layer connectivity through Si bridging tetrahedra weakens, allowing for an increase in the layer-to-layer distance (Figs. 5 ▸ and 7 ▸; Table 3 ▸) and possibly for a higher magnitude of random stacking faults. In addition, when an Si atom in *Q* ^3^ conformation is removed, the geometrical conformation of the corresponding remaining bridging *Q* ^2^ Si tetrahedron evolves, as the number of Si atoms coordinating the apical oxygen is reduced from two to one. Both a higher magnitude of the random stacking faults and a change in the coordination sphere of Si bridging tetrahedra would explain the chemical shift of the Si *Q* ^2^ environment (Fig. 4 ▸), which is thought to occur in the case of stacking disorder (Cong & Kirkpatrick, 1996*b* ▸) and probably results from a change in the Si—O—Si angle between connected Si tetrahedra (Magi *et al.*, 1984 ▸). Finally, the observed magnitude of the Si chain depolymerization is too low to account for the observed increase in the Ca/Si ratio. Indeed, as the proportion of *Q* ^1^ environment does not exceed 16.4 ± 2.1%, the Ca/Si ratio should be at most ∼0.7 if only layer Ca is considered. Thus, samples having a Ca/Si ratio ranging between 0.84 ± 0.03 and 0.86 ± 0.0.1 certainly contain interlayer Ca.

In the case of CSH 0.83–323 K (Ca/Si = 0.87 ± 0.02), the Si *Q* ^3^ environment is not detected, which indicates that layer-to-layer connectivity through bridging Si tetrahedra is lost. This is in accordance with the layer-to-layer distance of 12.3 Å, which is 0.5–0.8 Å higher than that observed in the three other samples having a comparable Ca/Si ratio but in which a *Q* ^3^ Si environment is detected (*i.e.* in which layer-to-layer connectivity through Si bridging tetrahedra remains). The proportion of Si *Q* ^2^ environment in this sample is 87.9 ± 2.0%, as compared to 53.6 ± 3.6% in CSH 0.6–443 K, whereas the proportion of Si *Q* ^1^ environment is 12.1 ± 1.0% as compared to 4.0 ± 1.8% in CSH 0.6–443 K. This means that depolymerization preferentially affected bridging Si tetrahedra that were in a *Q* ^3^ configuration. For the same reasons as stated above, this sample contains interlayer Ca which contributes to holding every layer parallel to each other.

As for CSH 0.83–323 K, none of the samples having a Ca/Si ratio higher than 0.87 ± 0.02 have an Si *Q* ^3^ environment. In these samples, the proportion of Si *Q* ^2^ environment decreases with the Ca/Si ratio, down to 32.0 ± 7.6% when Ca/Si ratio reaches 1.38 ± 0.03. The proportion of Si *Q* ^1^ environment follows the inverse trend, reaching a maximum of 68.1 ± 3.8% (Fig. 5 ▸ and Table 2 ▸). In CSH 1.4–323 K and CSH 1.5–443 K, the remains of the wollastonite-like Si chain are mainly built of paired Si tetrahedra (in a *Q* ^1^ configuration; Fig. 1 ▸), and the mean Si chain length is 3–5 (Table 2 ▸). Again, in order to explain the observed Ca/Si ratio of these samples, the presence of interlayer Ca (or of a discrete Ca-rich impurity) has to be considered. Indeed, a structure with all Si bridging tetrahedra omitted (*i.e.* all being in a *Q* ^1^ configuration) and no interlayer Ca would have a Ca/Si ratio of 1. Introducing interlayer Ca up to the maximum possible interlayer occupancy while avoiding sharing of hydration spheres (similarly to tobermorite MDO2 from the Urals or plombierite; Bonaccorsi *et al.*, 2005 ▸; Merlino *et al.*, 2001 ▸) and assuming that Ca can only occupy a single interlayer plane owing to the observed layer-to-layer distance (Fig. 7 ▸) leads to a Ca/Si ratio of 1.25. To reach a Ca/Si ratio of 1.5, it must be assumed that interlayer Ca atoms connect through their hydration sphere and form ‘layers’ that have many structural similarities with nanocrystalline portlandite (Girão *et al.*, 2010 ▸; Grangeon, Claret, Linard & Chiaberge, 2013 ▸; Richardson, 2014 ▸). These ‘layers’ can be understood as interstratified with a C–S–H layer structure having bridging Si tetrahedra omitted (Fig. 9 ▸; Garbev, Beuchle *et al.*, 2008 ▸; Garbev, Bornefeld *et al.*, 2008 ▸; Girão *et al.*, 2010 ▸; Grangeon, Claret, Linard & Chiaberge, 2013 ▸; Nonat, 2004 ▸; Richardson, 2014 ▸). This is compatible with the observed layer-to-layer distance reduction when the Ca/Si ratio increases from 0.87 ± 0.02 to 1.38 ± 0.03. Taking portlandite as a model structure, the typical height of a Ca(OH)_2_ octahedron is 2.3 Å (Busing & Levy, 1957 ▸), whereas two bridging Si tetrahedra connected through their apical oxygen have a height of 4.4 Å (Merlino *et al.*, 1999 ▸, 2001 ▸). In this assumption, the layer-to-layer distance can theoretically reduce down to ∼9.4 Å if all Si bridging tetrahedra are omitted and if interlayer Ca(OH)_2_ forms a ‘layer’ (Richardson, 2014 ▸). This was not observed in the presently studied samples, even at high Ca/Si ratios, because all samples still contain a few Si bridging tetrahedra which probably prevent the structure from collapsing.

The presence of Ca(OH)_2_ is in agreement with charge-balance calculations, as the observed Ca/Si ratios of CSH 1.4–323 K and CSH 1.5–443 K are higher than the values that could be reached assuming that the charge arising from a vacant Si tetrahedron is balanced by interlayer Ca (Ca/Si max in Table 2 ▸), meaning that Ca is in excess in the structure. It is also in agreement with TEM observations of intimate mixing of C–S–H and Ca(OH)_2_ (Richardson *et al.*, 2010 ▸), with studies which used thermogravimetry to show that samples having a Ca/Si higher than ∼1.25 contain a Ca-rich component which melts at the temperature expected for Ca(OH)_2_ (Kim *et al.*, 2013 ▸; Marty *et al.*, 2015 ▸), and with nano-indentation models (Chen *et al.*, 2010 ▸; Vandamme & Ulm, 2013 ▸). The presence of interlayer Ca subject to carbonation, such as a portlandite-like phase, would explain the observed preferential carbonation in samples of high Ca/Si ratio (present study; Yu *et al.*, 1999 ▸) and the XANES observation that samples of high Ca/Si ratio have a component that could be close in structure to portlandite (Fig. 2 ▸).

Previous studies have shown that the increase in sensitivity to carbonation with increasing Ca/Si ratio is much lower for wet samples, in which the infrared absorption band at ∼1350–1550 cm^−1^ is significant only for samples having a Ca/Si ratio higher than ∼1.4 (Walker *et al.*, 2007 ▸). This may indicate that the interlayer Ca(OH)_2_ would not exist in wet samples, perhaps because it dissolves preferentially when the sample is introduced into an aqueous medium [or, conversely, it could be interpreted as Ca(OH)_2_ structure formation in the interlayer space during water removal resulting from sample freeze-drying]. This hypothesis of a change in Ca distribution upon wetting (or drying) would explain the contrasting views from structure and chemical studies, where the former (performed on dried samples) suggest the presence of Ca(OH)_2_ (*e.g.* Chen *et al.*, 2010 ▸; Girão *et al.*, 2010 ▸; Richardson, 2014 ▸) while the latter (performed on aqueous suspensions) suggest that Ca accumulates at the particle surface through physical adsorption, thus explaining the observed charge inversion as a function of pH conditions (*e.g.* Labbez *et al.*, 2006 ▸, 2007 ▸). Such a hypothesis is consistent with the fact that, when C–S–H is immersed in a solution containing sodium, this cation is in the diffuse ion swarm, whereas it forms an outer-sphere complex, possibly in the C–S–H interlayer, when the sample is dried (Viallis *et al.*, 1999 ▸).

### 4.2. Use of X-ray diffraction to study C–S–H structure   

C–S–H XRD patterns are sometimes considered to be typical of an ‘X-ray amorphous’ phase (*e.g.* Kulik & Kersten, 2001 ▸; Nonat, 2004 ▸), perhaps because they contain only a few broad and weak diffraction maxima and because, in contrast to other nanocrystalline turbostratic structures (Grangeon *et al.*, 2014 ▸), modification of the XRD patterns upon variation of the chemical composition (here, Ca/Si ratio) is limited (Fig. 6 ▸). However, the present article, in combination with previous work, allows us to propose indicators for the qualitative and quantitative study of C–S–H structure.

First, it is proposed for the first time that the occupancy of bridging Si sites in the wollastonite-like chains can be estimated from the study of the maximum at ∼16.1° 2θ Cu *K*α (Fig. 8 ▸). Second, the 001 reflection whose position generally varies between 6.5 and 8.0° 2θ Cu *K*α can be exploited in two different manners: First, its breadth is indicative of the crystallite size along c* (mean number of layers stacked parallel to each other); the larger the breadth, the lower the crystallite size, although other factors such as microstrains or interstratification play a role (Grangeon, Claret, Linard & Chiaberge, 2013 ▸). Second, its position might be used as a proxy for the layer-to-layer distance (if the breadth of the reflection remains constant), but this has to be used with care. If the layer-to-layer distance is homogeneous in the crystallites forming the sample and if the crystallite size along c* is about constant, then the observed position of the 001 reflection is a function of the layer-to-layer distance. However, in the case of C–S–H, interstratification and (or) variation of crystallite size as a function of Ca/Si ratio may exist (Grangeon, Claret, Linard & Chiaberge, 2013 ▸). Consequently, quantitative analysis of the 00*l* reflections is recommended. Finally, the crystallite size in the *ab* plane (*i.e.* lateral extension of C–S–H crystallites) can be probed through the study of the breadth of the two maxima at 29.3 and 30° 2θ Cu *K*α, which both broaden when the crystallite size in the *ab* plane decreases. The evolution of *a* can be estimated from the study of the position of the maximum at ∼30° 2θ Cu *K*α, which shifts towards higher *d* spacing (low-angle side) when *a* increases. The evolution of *b* can be estimated using the same methodology and the study of the maximum at ∼29.3° 2θ Cu *K*α.

Overall, the study of C–S–H diffraction patterns in the reciprocal lattice, as done here and previously on other (nanocrystalline) turbostratic phases such as clays (Gates *et al.*, 2002 ▸), carbon black (Warren, 1941 ▸), Mn oxides (Grangeon *et al.*, 2012 ▸) and Fe oxides (Hadi *et al.*, 2014 ▸), appears to be a traditional yet promising way to study C–S–H structure. Additional information may be obtained from the study of XRD patterns in real space, using high-energy X-ray scattering and analysis in the pair distribution function formalism (Skinner *et al.*, 2010 ▸; Soyer-Uzun *et al.*, 2012 ▸), which allows one to attenuate the effect of the intrinsic structural disorder (*e.g*. nanocrystallinity and turbostratism) reigning in C–S–H, as also demonstrated for nanocrystalline Mn oxides (Manceau *et al.*, 2013 ▸; Grangeon *et al.*, 2015 ▸).

## 5. Conclusion   

The present study aimed to contribute to a better understanding of C–S–H structure, when its Ca/Si ratio varies from ∼0.6 to ∼1.5. At low Ca/Si ratio, C–S–H is a turbostratic nanocrystalline tobermorite having, to the precision of the methods used, defect-free Si chains. When the Ca/Si ratio increases, the main structure evolution is the progressive depolymerization of Si chains through progressive omission of Si bridging tetrahedra and the progressive incorporation of interlayer Ca to compensate for the resulting layer charge. At the highest Ca/Si ratios, interlayer Ca may polymerize, possibly in the interlayer space, to form a structure that has many structural similarities with Ca(OH)_2_. Formation of a Ca(OH)_2_ phase is supported by the fact that Ca is in excess in the structure.

It has been demonstrated that modelling of powder XRD patterns may be used to quantitatively determine structure parameters such as crystallite size or layer-to-layer distance. In addition, it is proposed that the occupancy of Si bridging tetrahedra can also be estimated from analysis of XRD patterns through the study of the modulation at ∼16.1° 2θ Cu *K*α. In particular, a new method, based solely on the qualitative examination of the patterns, has been established and proved to be consistent with available literature data. Further studies, using independent methods of analysis, are required to consolidate these results.

To conclude, it must be remembered that this study was performed on freeze-dried samples. However, C–S–H is a nanocrystalline lamellar structure. As such, it has external (surface) and internal (interlayer) adsorption sites, to which calcium can attach. By analogy with clay minerals, water certainly has an impact on C–S–H structure, in particular through a change in the calcium hydration sphere. This is supported by the fact that water has an influence on Portland cement pastes (Feldman & Sereda, 1968 ▸). However, the potential influence of hydration/dehydration cycles on C–S–H structure remains to be investigated.

## Supplementary Material

XRD and TEM analysis of the tobermorite reference. DOI: 10.1107/S1600576716003885/po5053sup1.pdf

## Figures and Tables

**Table 1:** Sample synthesis temperature, and target and actual Ca/Si ratios

Sample		Target Ca/Si (atom atom^−1^)	Synthesis temperature (K)	Ca/Si ratio (atom atom^−1^)	*n* †
CSH 0.6–323 K		0.6	323	0.57 ± 0.05	59
CSH 0.6–443 K		0.6	443	0.61 ± 0.02	50
CSH 0.83–RT		0.83	Room temperature	0.84 ± 0.03	48
CSH 0.83–323 K		0.83	323	0.87 ± 0.02	47
CSH 0.83–353 K		0.83	353	0.86 ± 0.01	50
CSH 0.83–443 K		0.83	443	0.84 ± 0.02	49
CSH 1.0–323 K		1	323	1.04 ± 0.03	59
CSH 1.0–443 K		1	443	1.04 ± 0.05‡	1
CSH 1.4–323 K		1.4	323	1.38 ± 0.03	60
CSH 1.5–443 K		1.5	443	1.47 ± 0.04	60

† Number of independent EPMA analyses.

‡ Determined using X-ray fluorescence using an aliquot of the bulk sample. Uncertainty is estimated from measurement reproducibility.

**Table 2:** Relative abundance of the different ^29^Si sites retrieved from analysis of ^29^Si MAS NMR data, mean Si chain length, and maximum Ca/Si ratio that could be reached if the charge resulting from all vacant Si tetrahedra was compensated for by interlayer Ca (Ca/Si max) n.d. stands for ‘not detected’.

	Relative abundance of the different ^29^Si sites (%)				
Sample	*Q* ^1^	*Q* ^2^	*Q* ^3^	Mean chain length (number of Si tetrahedra)	Ca/Si max†
CSH 0.6–323 K	4.2 ± 1.5	55.6 ± 3.0	40.2 ± 1.5	48	1.02
CSH 0.6–443 K	4.0 ± 1.8	53.6 ± 3.6	42.4 ± 1.8	50	1.02
CSH 0.83–RT	16.4 ± 2.1	78.3 ± 4.2	5.3 ± 2.1	12	1.08
CSH 0.83–323 K	12.1 ± 1.0	87.9 ± 2.0	n.d.	17	1.06
CSH 0.83–353 K	9.4 ± 1.0	80.2 ± 2.0	10.5 ± 1.0	21	1.05
CSH 0.83–443 K	11.0 ± 1.8	77.8 ± 3.6	11.1 ± 1.8	18	1.06
CSH 1.0–323 K	40.8 ± 5.3	59.2 ± 10.6	n.d.	5	1.20
CSH 1.0–443 K	26.0 ± 4.8	74.0 ± 9.6	n.d.	8	1.13
CSH 1.4–323 K	68.1 ± 3.8	32.0 ± 7.6	n.d.	3	1.34
CSH 1.5–443 K	39.5 ± 6.2	60.4 ± 12.4	n.d.	5	1.20

† Maximum Ca/Si ratio that could be reached assuming that the charge originating from a vacant Si site is compensated for by interlayer Ca (Richardson, 2014 ▸).

**Table 3:** Crystal data derived from analysis of the 001 reflection *R*_wp_, *R*_exp_ and GoF were calculated over the 2–15° 2θ Cu *K*α angular range.

Sample	Layer-to-layer distance (Å)	Mean number of layers stacked coherently	*R*_wp_ (%)	R_exp_ (%)	GoF
CSH 0.83–RT	11.75	3.7	6.08	3.25	3.51
CSH 0.83–323 K	12.25	3.3	6.02	3.21	3.53
CSH 0.83–353 K	11.75	3.4	6.59	3.21	4.21
CSH 0.83–443 K	11.50	3.5	7.35	3.26	5.10
CSH 1.0–323 K	11.50	4.2	8.75	3.24	7.28
CSH 1.4–323 K	11.00	4.1	8.76	3.30	7.06
