# Supplementary material for: Structure of nanocrystalline calcium silicate hydrates: insights from X-ray diffraction, synchrotron X-ray absorption and nuclear magnetic resonance
Source: J Appl Crystallogr. 2016 Apr 12;49(Pt 3):771–83. doi: 10.1107/S1600576716003885 (PMC4886978; doi:10.1107/S1600576716003885)
Supplement: Supplementary file 1 [file j-49-00771-sup1.pdf]

JOURNAL OF
APPLIED
CRYSTALLOGRAPHY

Volume 49 (2016)

Supporting information for article:

Structure of nanocrystalline calcium silicate hydrates: insights from X-ray diffraction, synchrotron X-ray absorption and nuclear magnetic resonance

Sylvain Grangeon, Francis Claret, Cédric Roosz, Tsutomu Sato, Stéphane Gaboreau and Yannick Linard

Supplementary data to the article:

Structure of nanocrystalline calcium silicate hydrates: insights from X-ray diffraction, synchrotron X-ray absorption and nuclear magnetic resonance.

Authored by: Sylvain Grangeon, Francis Claret, Cédric Roosz, Tsutomu Sato, Stéphane Gaboreau and Yannick Linard

Methods

Transmission electron microscopy (TEM) was performed using a Philips CM 20 operated at 200 kV. Synthetic tobermorite was dispersed in ultra-pure ethanol and then deposited on carbon-coated copper grids that were used for TEM analysis.

Figure S1. X-ray diffraction pattern of the synthetic tobermorite sample used for the present study (main panel) and typical crystal morphology, as seen by TEM (inset). Layer-to-layer distance of this synthetic tobermorite is 11.3 Å (Grangeon *et al.*, 2013).

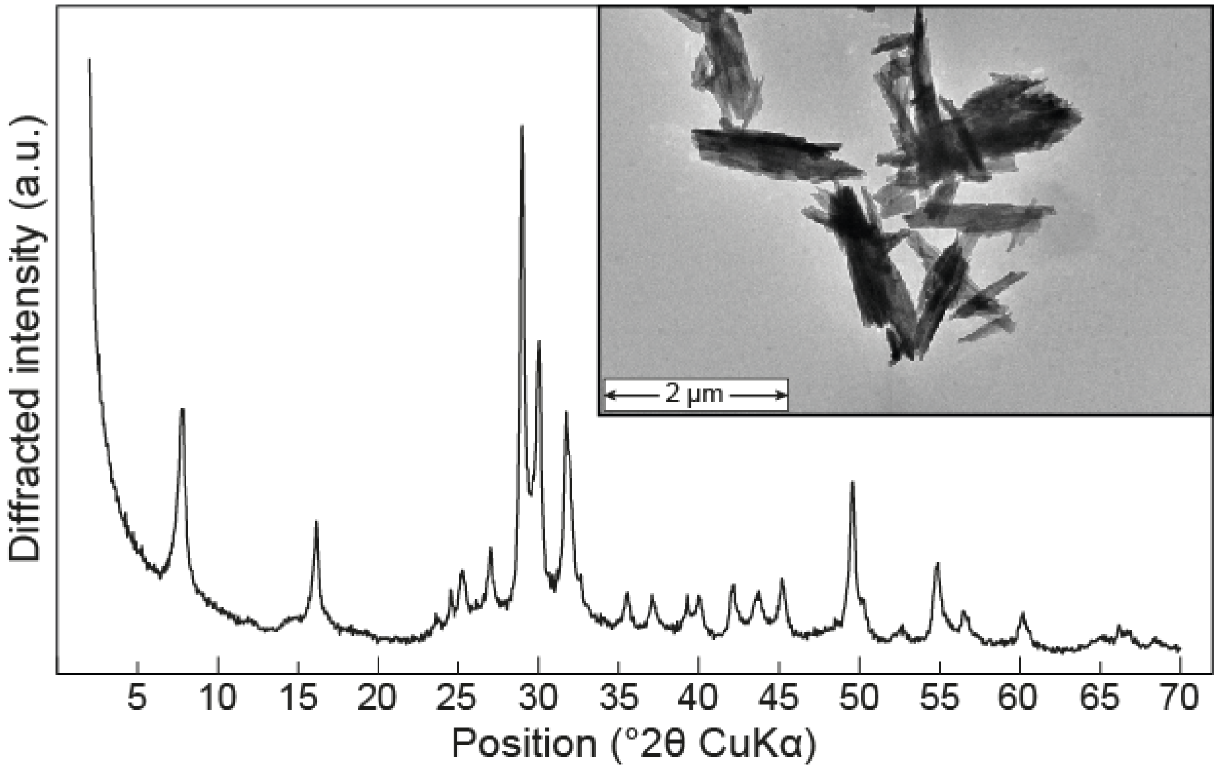

References

Grangeon, S., Claret, F., Lerouge, C., Warmont, F., Sato, T., Anraku, S., Numako, C., Linard, Y. & Lanson, B. (2013). *Cem. Concr. Res.* **52**, 31-37.
